# Targeting argininosuccinate synthetase negative melanomas using combination of arginine degrading enzyme and cisplatin

**DOI:** 10.18632/oncotarget.3370

**Published:** 2015-01-31

**Authors:** Niramol Savaraj, Chunjing Wu, Ying-Ying Li, Medhi Wangpaichitr, Min You, John Bomalaski, Wei He, Macus Tien Kuo, Lynn G. Feun

**Affiliations:** ^1^ Miami VA Healthcare System, Department of Veterans Affairs, Miami, FL, USA; ^2^ Department of Medicine, University of Miami, Miller School of Medicine, Miami, FL, USA; ^3^ Department of Surgery, University of Miami, Miller School of Medicine, Miami, FL, USA; ^4^ Polaris Group, San Diego, CA, USA; ^5^ Departments of Molecular Pathology, MD Anderson Cancer Center, Houston, TX, USA

**Keywords:** Melanoma, Cisplatin, Arginine Deiminase, Argininosuccinate Synthetase

## Abstract

Loss of argininosuccinate synthetase (ASS) expression in melanoma makes these tumor cells vulnerable to arginine deprivation. Pegylated arginine deiminase (ADI-PEG20) which degrades arginine to citrulline and ammonia has been used clinically and partial responses and stable disease have been noted with minimal toxicity. In order to improve the therapeutic efficacy of ADI-PEG20, we have combined ADI-PEG20 with a DNA damaging agent, cisplatin. We have shown that the combination of the two drugs together significantly improved the therapeutic efficacy when compared to ADI-PEG20 alone or cisplatin alone in 4 melanoma cell lines, regardless of their BRAF mutation. *In-vivo* study also exhibited the same effect as *in-vitro* with no added toxicity to either agent alone. The underlying mechanism is complex, but increased DNA damage upon arginine deprivation due to decreased DNA repair proteins, FANCD2, ATM, and CHK1/2 most likely leads to increased apoptosis. This action is further intensified by increased proapoptotic protein, NOXA, and decreased antiapoptotic proteins, SURVIVIN, BCL2 and XIAP. The autophagic process which protects cells from apoptosis upon ADI-PEG20 treatment also dampens upon cisplatin administration. Thus, the combination of arginine deprivation and cisplatin function in concert to kill tumor cells which do not express ASS without added toxicity to normal cells.

## INTRODUCTION

We have previously shown that approximately 70% of melanoma tumors do not express argininosuccinate synthetase (ASS) and cannot synthesize arginine from citrulline. Therefore, arginine becomes an essential amino acid. Indeed, we and many investigators have shown that degrading arginine using either arginine deiminase (ADI) or arginase, both *in vivo* and *in vitro*, can lead to tumor inhibition and cell death [1-5]. Pegylated ADI (ADI-PEG20) has been tested in clinical trials and antitumor activity was seen in melanoma patients [6-8]. Furthermore, we have shown that the response is seen primarily in melanoma patients whose tumors do not express ASS (ASS(−)) [6]. However, we have found that tumor cells can undergo autophagy during arginine deprivation as a means to evade cell death. During this time, certain melanoma cells can turn on the ASS gene and become resistant to ADI-PEG20 treatment [5, 9-12]. Therefore, in order to increase the efficacy of ADI-PEG20, combination with other agent(s) is needed to evade autophagy and re-direct the cells toward apoptosis. Consequently, the antitumor response may be increased. Two well defined pathways regulate the execution of apoptosis: i) the intrinsic or mitochondria-initiated pathway and ii) the extrinsic or receptor-mediated pathway. The intrinsic pathway is caused by disruption of mitochondria membrane which is controlled by members of the BCL2 superfamily. Previously, we have shown that the addition of TRAIL leads to an increase in caspase 8 and cleavage of tBID and increases the apoptotic cell death upon arginine deprivation. Furthermore, activation of caspase 8 also can halt the autophagic process by cleavage of key apoptotic proteins, such as Beclin 1 and ATG5 [13-15]. Since TRAIL is not an FDA approved drug, we have studied other agents which can also activate the apoptotic process via the intrinsic pathway and determined the possible enhancement effect with ADI-PEG20. We have chosen cisplatin, one of the most widely used chemotherapeutic agents in solid tumors, which is known to trigger apoptosis via the intrinsic pathway[16]. We have found that combination of both agents increased the apoptosis without added toxicity. We report the results herein.

## RESULTS

### Increased growth inhibitory effect of cisplatin with ADI-PEG20

**Fig. 1:**
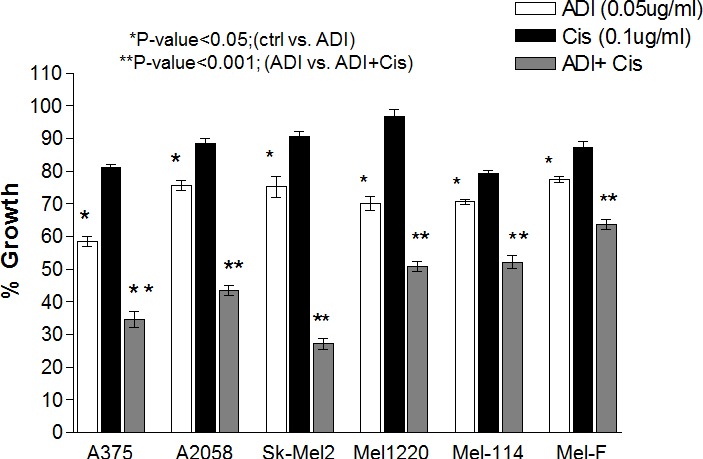
Growth inhibitory effects in 4 melanoma cell lines (A375, Sk-Mel2, A2058 and Mel1220 and 2 primary cultures ( Mel-114 and Mel-F) Cells were treated with ADI-PEG20 alone at 0.05μg/ml, or cisplatin alone at 0.1μg/ml, or in combination for 72hr. The untreated control was set at 100%. ADI-PEG20 alone results in approximately 60-77% viable cells depending on the cell lines and is statistically significant compared to control (P<0.05). Cisplatin at 0.1μg/ml results in 80-90% viable cells or less than 20% growth inhibition. Combination of ADI-PEG20+cisplatin results in 35-60% viable cells. Compared to ADI-PEG 20 alone, it is statistically significant (P < 0.001). The growth inhibitory effect was an average of three separate experiments with each experiment performed in duplicate.

Cells were treated with ADI-PEG20 (0.05μg/ml) alone or cisplatin (0.1μg/ml) alone or in combination for 72 hr. These concentrations are achievable for both drugs clinically. At 72 hr., viable calls were counted using trypan blue exclusion test. The growth inhibitory effect in 4 cell lines and 2 primary cultures were shown in Fig. 1. Cisplatin at this dosage showed less than 20% growth inhibition in all cell lines, while ADI-PEG20 alone resulted in 25-40% growth inhibition (Fig. 1). Combination treatment significantly increased growth inhibition in all ASS negative cells when compared to single agent alone (p<0.001). Moreover, we did not observe significant potentiation effect of ADI-PEG 20 combined with cisplatin in ASS positive cells (A2058R and Mel-GP). The ID_50_ of cisplatin was 0.25 μg/ml and 0.3 μg/ml, respectively with or without ADI-PEG20. Furthermore, ADI-PEG20 at 0.05 μg/ml had no effect on cell growth for both cell lines. This is expected due to the fact that these cells can synthesize arginine from citrulline. Overall, the addition of ADI-PEG20 increases the growth inhibitory effect of cisplatin in ASS(−) cells which indicates that arginine deprivation is an important factor responsible for this effect.

### Increased apoptotic effect of cisplatin upon concurrent treatment with ADI-PEG 20

**Fig. 2:**
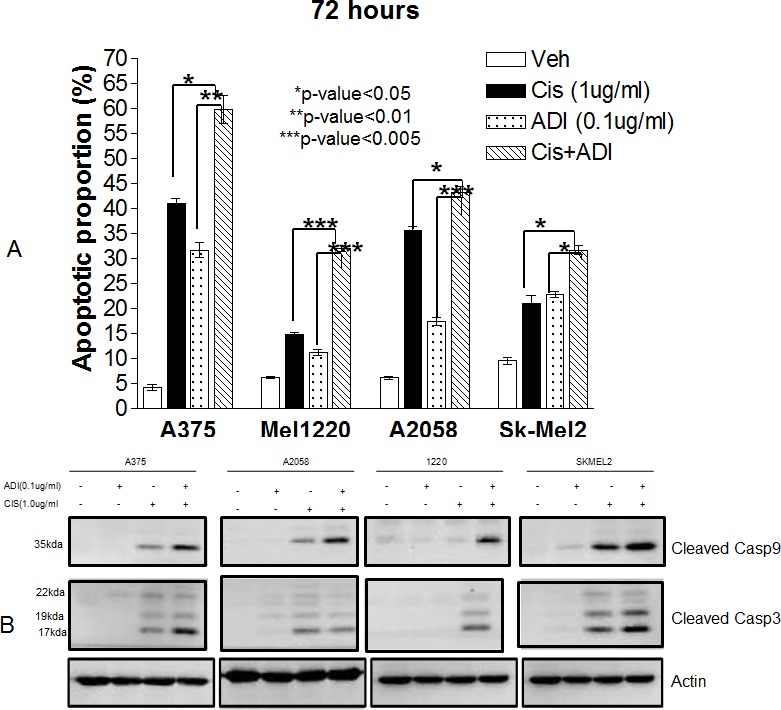
Apoptotic effect in 4 melanoma cell lines (A375, Sk-Mel2, A2058, and Mel1220) treated with ADI-PEG20 alone (0.1μg/ml), cisplatin alone (1μg/ml), and in combination for 72hr (A) The percentages of cell death as measured by annexin-V and PI. Compared with cisplatin, combination treatment statistically increased cell death in all cell lines with p<0.05 in 3 cell lines (A-375, A2058 and Sk-Mel2) and p<0.005 in Mel1220. Compared with ADI-PEG20 alone, combination treatment also statistically improved the percentage of cell death (p<0.01 in A-375, p<0.005 in Mel1220and A2058, and p< 0.05 in Sk-Mel2). (B) Illustrates cleaved caspase 9 in 4 cell lines when treated with ADI-PEG20 alone, cisplatin alone, and in combination. Combination treatment again showed higher amounts of cleaved caspase 9 & 3 when compared to either treatment alone. It also illustrates that the mode of cell death is via apoptosis. Actin was used as loading control.

We then investigated whether this combination also results in increased apoptotic cell death. Four ASS(−) cell lines (A375, Mel1220, A2058 and Sk-Mel2) and one ASS(+) cell line (Mel-GP) were used for this study. Cells were treated with ADI-PEG20 at 0.1μg/ml or cisplatin at 1μg/ml or both for 72 hr. The higher concentration of ADI-PEG20 was used in order to deplete arginine more rapidly and hence cells would be exposed to arginine-free environment for a longer period of time which enabled us to detect early apoptosis. Nevertheless, the concentration for both ADI-PEG20 and cisplatin are achievable clinically [16]. At 72 hr, apoptotic assay was performed using Annexin-V and PI. The results were shown in Fig. 2A and Fig. S1a. Combination treatment resulted in a significant increase in apoptotic cell death compared to either cisplatin alone or with ADI-PEG20 alone in all 4 ASS(−) cell lines, using the student t-test *(p-values* range from less than 0.005 to 0.05). This was not observed in ASS(+) Mel-GP cells. Fig. S1b showed that there was no change for cisplatin treatment alone vs. combination in Mel-GP (29.3% vs.27.8%, respectively). To determine that the apoptosis was caspase mediated, we have assayed caspase 3 and 9 by Western blot. The data were shown in Fig. 2B. All 4 ASS (−) cell lines exhibited an increase in cleaved caspase 9 and caspase 3 when cells were exposed to both drugs. In Mel1220, the increase in cleaved caspase 9 and 3 was not well visualized when treated with cisplatin alone; however, it was well visualized with the combination. To further confirm that apoptosis was mediated by caspase, we have co-treated A2058 cells with pan-caspase inhibitor Z-VAD-FMK and this was able to reverse the apoptotic process (see Fig. S2). Thus, our data strongly indicate that the addition of ADI-PEG20 significantly increased the antitumor effect of cisplatin in ASS (−) melanoma cell lines, but not in ASS (+) cells.

### Augmentation of antitumor effect of ADI-PEG 20 is also seen in xenograft

**Fig. 3:**
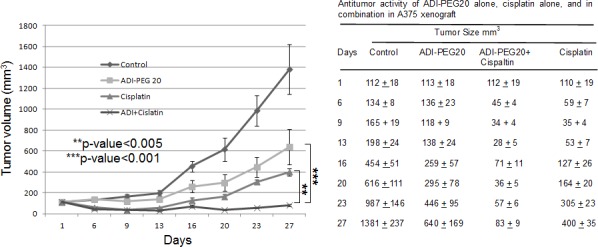
*In-vivo* antitumor activity of ADI-PEG20 alone, cisplatin alone, and in combination in A375 xenograft Tumor volume was measured prior to treatment as stated in the Methods section. Compared to vehicle control, all three treatment groups showed statistically better results than control group (p<0.001). Compared with ADI-PEG20 alone, combination treatment is highly statistically superior to ADI-PEG20 with p<0.001. Combination also showed highly statistically better results than cisplatin alone with p<0.005. Table shows the actual tumor measurement of ADI-PEG20 alone, cisplatin alone, and in combination in A375 xenograft.

Mice were given ADI-PEG20 IM alone or cisplatin IP alone or in combination as stated in the method section. The results of the tumor growth curves and tumor sizes at different time points after initiation of therapy were shown in Fig. 3. The mean tumor size of saline treated control mice reached 1381 mm^3^ at 27 days after treatment. Treatment with ADI-PEG20 alone (53.3IU/kg, q6d x 4) delayed tumor growth by 7 days at 400 mm^3^ size, and produced a mean tumor size of 640 mm^3^ size at day 27 (T/C value = 46%, p < 0.001) compared with control group. Treatment with cisplatin alone (6mg/kg, q6d x 3) delayed tumor growth by 12 days at 400 mm^3^ size, and produced a mean tumor size of 400 mm^3^ on day 27 (T/C value = 29%, p < 0.001) compared with control group. Compared with the treatment of ADI-PEG20 alone or cisplatin alone, the combination of the two drugs further delayed the tumor growth by day 27, with a mean tumor size of 83 mm^3^ (T/C value = 6% p < 0.001 compared to ADI-PEG20 and p<0.005 compared to cisplatin alone), respectively. Thus, both our *in-vitro* and *in-vivo* data strongly indicated that the combination of cisplatin and ADI-PEG20 significantly reduced the tumor size compared with either agent alone. The toxicity in mice was not increased and there were no deaths from the treatment. Although temporary body weight loss (10%) occurred immediately after each treatment, the body weight recovered to normal before each treatment.

### Mechanism of increased antitumor effect of cisplatin by ADI-PEG20

**Fig. 4:**
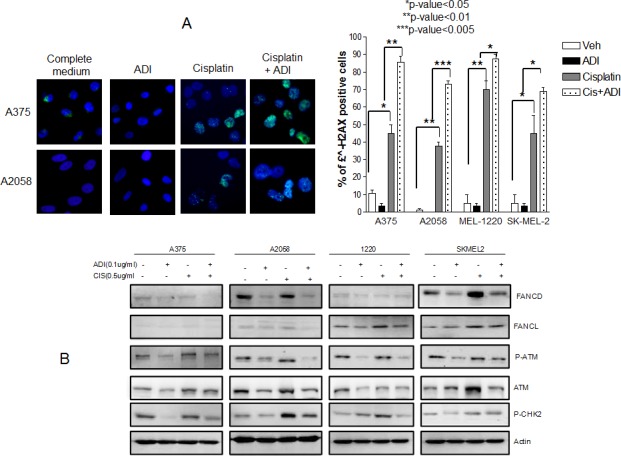
Increased DNA damage in 4 cell lines when treated with combination treatment (A) Immunofluorescence of γH2AX foci after treatment with cisplatin at 0.5 μg/ml for 48 hr in normal media and in ADI-PEG20 treated media. The γH2AX foci increased in all 4 cell lines with cisplatin alone (p≤0.05 in A375 and SkMel2, p≤0.01 in A2058 and Mel1220) which was expected since cisplatin is known to cause DNA damage. Upon arginine deprivation alone, there were no changes in γH2AX foci compared to control; however, the γH2AX foci increased significantly on combination treatment in all 4 cell lines, which indicates that in the absence of arginine, cisplatin treatment results in more damage (p<0.01 in A-375, p<0.005 in A-2058, p<0.05 in Mel-1220 and Sk-Mel2). (B) Illustrates that increase in DNA damage is secondary to decrease in DNA repair process. Ubiquitinated FANCD, p-ATM, ATM, and p-CHK2, which increased in response to cisplatin treatment to initiate the repair process, showed a decrease upon combination treatment. The amount of FANCL also slightly decreased upon combination treatment in 3 cell lines. A375 possess very low amounts of FANCL and so differences could not be visualized.

Enhanced DNA damage and decreased DNA repair protein are seen in melanoma cells treated with cisplatin and ADI-PEG20. It is known that cisplatin treatment results in DNA damage which causes cell cycle arrest and eventually cell death. In order for cells to survive, DNA repair must occur [17, 18]. We hypothesize that arginine deprivation will impair the repair process and hence increase DNA damage which drives the cells toward apoptosis. We have treated all 4 cell lines with cisplatin alone in normal media or in ADI-PEG20 treated media for 48 hr. The extent of DNA damage was assessed by examining the foci of γH2AX, a marker for double strand DNA break [19]. Treatment with cisplatin showed an increase in γH2AX compared to control (p<0.05 for A375 and Sk-Mel-2 and p<0.01 for A2058 and Mel1220). ADI-PEG20 treatment showed no significant change compared to control. However, with ADI-PEG20 and cisplatin, all 4 cell lines showed a statistical increase in γH2AX when compared to cisplatin in normal media (p<0.01 for A375, p<0.005 for A2058,and p<0.05 for both Mel1220 and Sk-Mel2) (Fig. 4A). Thus, our results suggested that the addition of ADI-PEG20 to deplete the arginine in the media with cisplatin results in an increase in DNA damage, most likely secondary to impairment of DNA repair. To elucidate which proteins were involved, we have performed cDNA Microarray after cells were treated with ADI-PEG20 for three days in three cell lines, A2058, A375, and Mel1220. We have found that FANCD2 (accession NM_033084) was consistently lower in all three cell lines after ADI-PEG20 treatment, while FANCL (accession NM_018062) showed less than 2 fold decrease in all cell lines(data not shown). There were no changes in ATR, ERCC family and BRCA family, RAD50, RAD51 and RPA. It is known that both ATM and FANCD are important proteins to initiate repair of DNA double strand breaks. Thus, a decrease of these two proteins can lead to impairment of DNA repair process. To confirm this, we have performed immunoblot of ATM, FANCD2, and FANCL in 4 cells lines after treatment with ADI-PEG20 alone, cisplatin alone, and in combination. The results were shown in Fig. 4B. ATM and Phospho-ATM were consistently lower in 4 cell lines after treatment with ADI-PEG20 alone when compared to control, and increased after exposure to cisplatin in response to DNA damage and decreased again upon combination treatment (Fig. 4B). Phosphor-Check2 (CHK2), a cell cycle checkpoint regulator known to be phosphorylated by ATM also decreased after ADI-PEG20 treatment, increased after cisplatin treatment and again decrease after combination. It is known that Fanconi anemia (FA) family is essential for repairing DNA crosslinks [17, 20]. Monoubiquitination of FANCD2 is essential for orchestrating the whole DNA repair process which involves many family members of FA including FANCL [21-23]. Decreased FANCD2 was seen with ADI-PEG20 alone, and increased after cisplatin treatment in response to DNA damage. This protein also decreased after combination treatment. Overall, arginine deprivation alone resulted in a decrease in multiple DNA repair proteins, but did not cause DNA damage. On the other hand, cisplatin treatment alone resulted in DNA damage which triggered an increase in DNA repair proteins. Importantly, with the combination, the lower levels of DNA repair proteins produced by arginine deprivation still persisted and was not completely negated by the induction of DNA repair proteins caused by cisplatin treatment. Thus, the net result still showed lower DNA repair proteins and hence an increase in DNA damage as illustrated by an increase in γH2AX foci. In contrast, for A2058R cell line which express ASS, treatment with ADI-PEG20 alone did not have any effect in FANCD2 and phosphor-ATM and hence there were no changes in these proteins upon combination treatment when compared to cisplatin alone (Fig. S3). As a result, there were no change in the growth inhibitory or apoptotic effect

### Interference with apoptotic and anti-apoptotic proteins by ADI-PEG20 enhances the antitumor effect to cisplatin

**Fig. 5:**
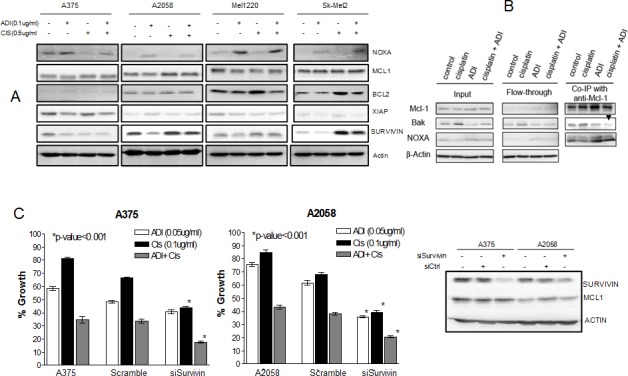
Illustrates the involvement of apoptotic pathway in combination treatment (A) Cells were treated with ADI-PEG20 at 0.1μg/ml or cisplatin 0.5μg/ml or both for 72 hr. then lysed and immunoblotted with pro-and anti apoptotic protein. Treatment with ADI-PEG20 results in increase in NOXA in all 4 cells while treatment with cisplatin results in a decrease in NOXA. The increase in NOXA persist upon combination treatment with (see supplement for quantification). There was no change in BCL2 after ADI-PEG20 treatment, but BCL2 increased after cisplatin treatment and decreased after combination treatment. XIAP decreased after ADI-PEG20 treatment in A375, increased after cisplatin and again decreased after combination. SURVIVIN decreased in all 4 cell lines after ADI-PEG20 and increased after cisplatin treatment and again decreased upon combination treatment. Actin was used as control. (B) Immunoprecipitate using MCL1 in A2058 after cisplatin treatment alone, ADI-PEG20 alone and in combination treatment. More NOXA binding to MCL-1 was seen after ADI-PEG20 treatment while increased BAK binding to MCL1 was seen after cisplatin treatment. Upon combination treatment, increased NOXA binding to MCL-1 occurred and much less BAK was bound to MCL1(Black arrow head). This unbound BAK was able to induce MOMP. (C) Silencing SURVIVIN in A375 and A2058 cell lines resulted in >90% decrease in SURVIVIN, both mRNA (data not shown) and protein levels. There was no effect in MCL1 (control) and actin was used as internal control. Silencing SURVIVIN resulted in significantly increased sensitivity to cisplatin alone, and in combination for both cell lines (P<0.001).

Another factor which can contribute to the demise of melanoma cells is the alteration in the pro- and anti-apoptotic proteins which favors apoptosis. We have previously shown that an increase in proapoptotic protein and decrease in antiapoptotic protein occurs upon arginine deprivation [13, 14]. However, the signal is not sufficiently intense to drive the cells toward apoptosis and instead the cells undergo autophagy as the mean for survival [9]. In contrast, exposure to cisplatin results in upregulation of antiapoptotic protein to evade apoptotic cell death from DNA damage [16, 24, 25]. We hypothesize that combination treatment with ADI-PEG20 and cisplatin results in the inability of cells to up-regulate antiapoptotic proteins and hence lead to an increase in cell death. In this context, we have examined NOXA, MCL1, BCL2, XIAP, and SURVIVIN in 4 cell lines upon exposure to ADI-PEG20 alone, cisplatin alone and in combination. The pro and antiapoptotic proteins were chosen from our previous publications [13, 14] and cDNA microarray data. The proteins were assayed by immunoblot. The results were shown in Fig. 5A.

Treatment with ADI-PEG20 alone showed no significant change in BCL2, while cisplatin alone showed a slight increase in 3 cell lines (A2058, Mel1220 and Sk-Mel2) (Fig. 5A). A375 does not express BCL2. The combination showed a decrease in all 3 cell lines (A2058, Mel1220 and Sk-Mel2). Similar to BCL2, treatment with cisplatin also led to an increase in XIAP in A375 and Sk-Mel2 while combination treatment showed lower levels. However, there was no significant change in MCL1. It is known that NOXA (proapoptotic protein) can bind MCL1 and interfere with its antiapoptotic function [26, 27]. Our previous results as well as current results indicated that NOXA was increased in all 4 cell lines after treatment with ADI-PEG 20 (see Fig. S4 for band quantification). In contrast, exposure to cisplatin resulted in down regulation of NOXA while the combination increased NOXA (Fig. 5A and Fig. S4). Thus, it is possible that elevated NOXA levels upon arginine deprivation counter balance the decrease in NOXA seen upon cisplatin treatment. Since NOXA can bind MCL1, and MCL1 is known to binds BAK and prevent BAK to initiate the apoptosis, higher NOXA levels can displace BAK from binding MCL1 and hence more free BAK is available to initiate the apoptotic process. To determine this, we have performed immunoprecipitation with MCL1 and then detected the immunoprecipitate with NOXA and BAK. The results were shown in Fig. 5B. NOXA was associated with MCL1 after ADI-PEG20 treatment. The increase of NOXA association with MCL1 is due to the increased NOXA protein after ADI-PEG20 treatment as could be seen from the input. Cisplatin treatment alone decreased NOXA protein with subsequent less binding to MCL1. However, with the combination treatment, this trend was reversed by ADI-PEG20. The amount of NOXA bound to MCL1 was comparable to that after ADI-PEG20 treatment alone. Thus, with more NOXA bound to MCL1, there was less BAK associated with MCL1 (Fig. 5B, black arrow head). This is consistent with earlier reports that NOXA can displace the interaction of BAK with MCL1, thus freeing the BAK for initiation of apoptosis via increased mitochondria outer membrane permeabilization (MOMP). Although cisplatin alone increased the expression of BAK, the binding of BAK to MCL1 also increased and hence was unable to disrupt the mitochondrial outer membrane. Overall, ADI-PEG20 combined with cisplatin effectively reduced the amount of BAK bound to MCL1 due to an increase in NOXA binding to MCL1, and hence more free BAK was available to initiate the apoptotic process.

Treatment with ADI-PEG20 alone also showed down regulation of SURVIVIN. Upregulation of SURVIVIN has been shown in melanoma tumor samples and is one of the contributory factors to drug resistance [28]. Fig. 5A and Fig. S4 showed that treatment with cisplatin increased SURVIVIN, while combination showed an overall decrease. In order to further delineate the role of SURVIVIN with this combination treatment, we have knock down SURVIVIN using siRNA and examined the growth inhibitory effect of ADI-PEG20 alone, cisplatin alone and in combination. The results were shown in Fig. 5C. Silencing SURVIVIN showed a significant increase in antitumor effect with cisplatin alone and with the combination of cisplatin + ADI-PEG20 in both cell lines (p< 0.001). Importantly, comparing the growth inhibitory effect of cisplatin + ADI-PEG20 with cisplatin + knock down of SURVIVIN, the combination treatment of cisplatin + ADI-PEG20 had slightly better effect (35% VS 48%, respectively, in A375) than silencing SURVIVIN alone. This is expected since treatment with ADI-PEG20 can decrease other antiapoptotic proteins besides SURVIVIN. On the other hand, in A2058 cell line, there was no change in growth inhibitory effect between the silencing SURVIVIN with cisplatin versus ADI-PEG20 with cisplatin. It is possible that in A2058 cell line, SURVIVIN plays a major antiapoptotic role upon exposure to cisplatin. In addition, the level of SURVIVIN was much lower in the silencing condition than when the cells were treated with ADI-PEG20 alone. These two factors could explain the better therapeutic effect on silencing SURVIVIN compared to combination treatment. Overall, we can conclude that a decrease in antiapoptotic proteins and an increase in proapoptotic proteins by arginine deprivation contributes to the potentiation effect of ADI-PEG 20 and cisplatin seen in ASS(−) melanoma cell lines.

**Fig. 6:**
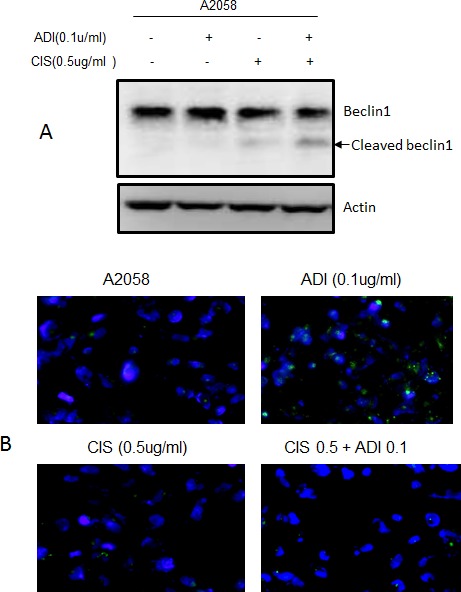
Arginine deprivation leads to autophagy was abrogated by the addition of cisplatin (A) Immunoblot of Beclin1 and its cleaved form after treatment with ADI-PEG20 alone, cisplatin alone, and in combination. Treatment with cisplatin alone resulted in slight cleavage of Beclin 1 while the combination resulted in higher increased in cleaved Beclin1. (B) Treatment with ADI-PEG20 alone resulted in autophagy as detected by increasing autophagic vacuoles indicated by green fluorescent dot while combination treatment showed much less autophagic vacuoles.

**Diagram 1:**
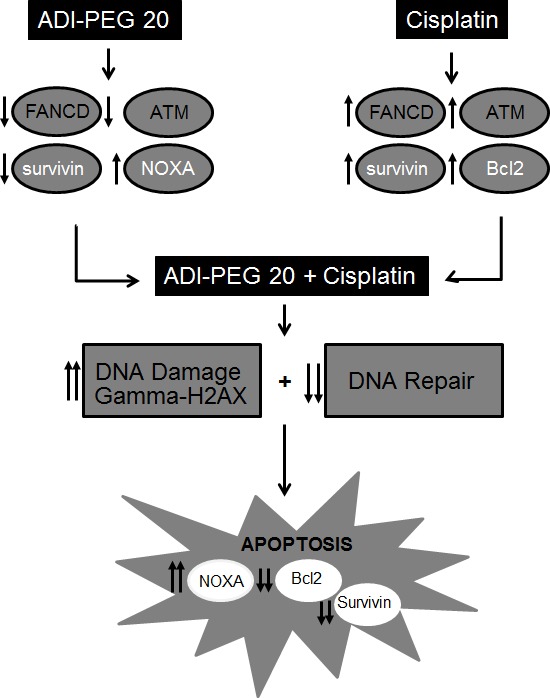
depicts the possible mechanism involved in this enhanced antitumor effect upon combination treatment

Taking together all the above data, the augmentation of the cytotoxic effect of cisplatin by ADI-PEG20 involves multiple mechanisms. Increased DNA damage secondary to decrease in DNA repair process triggers the stronger apoptotic signal, which is further enhanced by a decrease in antiapoptotic proteins and an increase in pro-apoptotic proteins. Hence, this drives the cells toward the apoptotic pathway (see Diagram 1). In addition, treatment with cisplatin also activates caspase cascade and cleavage of Beclin1 (Fig. 6A). This halts the autophagic process, a pro-survival mechanism upon ADI-PEG20 treatment due to arginine deprivation [9]. To further confirm this, we have assayed autophagy using the Cyto-ID autophagy detection kit from Enzo Life Science, as illustrated in Fig. 6B. ADI-PEG20 treatment alone showed multiple autophagic vacuoles which diminish upon combination treatment with cisplatin. Thus, by abrogating the survival mechanism upon arginine deprivation with ADI-PEG-20 treatment using cisplatin, one can re-direct the cells toward apoptosis. Overall, by combining arginine deprivation using ADI-PEG20 which is specific for ASS(−) melanoma tumor cells with cisplatin, one can significantly enhance the antitumor effect without an increase in toxicity as seen with other combination therapies.

## DISCUSSION

Substantial progress has been made for the treatment of malignant melanoma in the past two years. This includes the discovery of BRAF inhibitors (vemurafenib and dabrafenib) and MEK inhibitor (trametinab). This BRAF/MEK treatment is effective in tumors which carry the BRAF mutation. On the other hand, resistance is inevitable and side effects which include a high incidence of cutaneous squamous cell carcinoma [29-32] can occur. Another class of agents such as ipilimumab offers durable response with a response rate of 25% [33, 34]. However, treatment also results in significant side effects including colitis. We and others have found that certain melanomas do not express ASS, a key enzyme in the urea cycle for synthesizing arginine. Thus, arginine becomes an essential amino acid in this setting. This has led to the development of pegylated arginine deiminase (ADI-PEG20) which degrades arginine to citrulline and ammonia. Melanomas which lack ASS (ASS(−)) cannot synthesize arginine from citrulline and hence arginine deprivation therapy in these tumors results in growth inhibition and prolonged starvation leading to apoptosis. Besides ADI-PEG20, arginase, an enzyme present in human cells which degrades arginine to ornithine and urea, also has been shown to be active in ASS(−) tumors including melanoma [35, 36]. However, ornithine transcarbamylase (OTC) is needed to convert ornithine to citrulline. Thus, normal cells which lack OTC can become toxic while tumor cells which lack both ASS and OTC are more sensitive [36]. Nevertheless, its pegylated and modified form to improve activity and pharmacokinetic profile has been synthesized [37, 38] in preparation for clinical trial. Another factor which influences the response to arginine deprivation is autophagy which enables melanoma cells to survive and hence combination treatment is needed to enhance the apoptotic signal. In fact, we have found that combination of TRAIL with ADI-PEG20 leads to an increase in apoptosis. However, TRAIL is not clinically available. In this laboratory investigation, we used a combination ADI-PEG20 with cisplatin, a commonly used chemotherapeutic agent which shows antitumor activity against a wide variety of solid tumors. We have found that the combination of ADI-PEG20 with cisplatin significantly enhances the antitumor effect in all ASS negative melanoma cell lines. This enhanced effect was also demonstrated *in vivo* with mice and the combination was well tolerated with no added toxicity compared to cisplatin alone. This combination has moved to the clinic and is currently in phase I- II trial at M.D. Anderson Cancer Center (ClinicalTrials.gov Identifier: NCT01665183).

It is known that cisplatin treatment results in DNA crosslinking and possible translesional synthesis [17]. We have found that treatment with ADI-PEG20 in ASS(−) cells leads to a decrease in FANCD, an important DNA repair protein for cisplatin. FANCD2 belongs to the Fanconi Anemia family which has at least 15 family members [39]. DNA damage stimulates monoubiquitinylation of FANCD2 by the FA core complex, a multi-subunit E3 ubiquitin ligase [40]. Subsequently, this leads to DNA double breaks and activation of ATM which is also decreased upon arginine deprivation. This process is required for cells to survive cisplatin insult [41]. The key question is why arginine deprivation leads to decreased FANCD2. Recently, it has been shown that mTOR1-S6K1 signaling controls the transcription of FANCD2 [42, 43]. In this regard, we have previously shown that arginine deprivation by ADI-PEG20 leads to inhibition of mTOR [5] while cisplatin treatment can result in mTOR activation [9, 44]in an attempt to evade cell death. Thus, it is possible that inhibition of mTOR by arginine deprivation is a possible mechanism(s) why FANCD2 is decreased. To support this notion, we have assayed phosphor-p70S6K and phosphor 4E-BP, the two proteins known to be phosphorylated by mTOR. As shown in Fig. S5, arginine deprivation does lead to lesser 4E-BP and p70S6 kinase phosphorylation while cisplatin increases them and the combination treatment leads to lesser mTOR activation (less 4EBP phosphorylation and phosphorylation of p70S6K) when compared to cisplatin Thus, our data suggest that lower FANCD2 is most likely mediated via mTOR pathway, as previously reported by others.

The other mechanism which contributes to the potentiation of this combination is the alteration in the pro- and antiapoptotic proteins from arginine deprivation. Our data indicate that treatment with cisplatin can result in upregulation of antiapoptotic proteins in an attempt for the cell to evade apoptosis [45]. This effect is negated by increasing pro-apoptotic protein such as NOXA upon arginine deprivation. On the other hand, arginine deprivation leads to autophagy as its survival mechanism which was terminated by activation of caspase cascade by cisplatin which can cleave Beclin 1 and terminate the autophagic process.

Another possible mechanism of this elevated cytotoxic effect of cisplatin and ADI-PEG20 is mediated via nitric oxide (NO). It is known that arginine is the major substrate of NO. Thus, arginine deprivation undoubtedly leads to a decrease in NO (Fig. S6). This finding also has been previously reported in tissue culture, animals and humans with either melanoma or hepatocellular carcinoma [7, 46-48]. Depletion of NO can inhibit melanoma proliferation, and enhance cisplatin-induced apoptosis in melanoma cells in tissue culture [49, 50]. In addition, NO inhibition with the selective antagonist N6-(1-iminoethyl)–L-lysine dihydrochloride (L-NIL) synergized with cisplatin in xenograft models without added toxicity [50]. Interestingly, although the mice only received 3 doses of cisplatin every 3 days, continued dosing of L-NIL was sufficient to suppress tumor growth [50]. Also, endogenously produced NO mitigates sensitivity of melanoma cells to cisplatin [51]. Similarly, acquired resistance to cisplatin in lung cancer cells has been shown to be mediated by NO, and NO-mediated drug resistance was found to be reversible when cells were further cultured in the absence of NO [52]. Thus, it is possible that depletion of NO contributes to the increased sensitivity to cisplatin. On the other hand, NO is also known to have both cytostatic and cytocidal effects against tumor cells, and nitric oxide donor has been shown to sensitize tumor cells to radiation [53]. P53 status also plays a key factor to the dichotomy effect of NO [53]. Overall, the paradoxical effect of NO most likely depends on the tumor microenvironment, immune system, oncogene and tumor suppressor genes. Whether sensitization of cisplatin by ADI-PEG20 in melanoma cells is partly mediated by NO is not proven at this time and may depend on molecular genetic alteration of each cell line and tumor microenvironment.

In summary, we have found an effective combination to treat malignant melanomas which do not express ASS, regardless of their BRAF mutation status. Since ADI-PEG20 is well tolerated, this combination of ADI-PEG20 with cisplatin which drive the cells toward apoptosis, is feasible. Other combinations such as with temozolomide and taxotere also have been examined; however, the enhancement effect is not as significant as with cisplatin in melanoma [54]. In this study, we have clearly shown that combination of cisplatin with ADI-PEG20 is superior to either treatment alone and does not produce overt toxicity *in vivo*. The mechanism(s) involved is complex and appears to involve multiple mechanisms. All these mechanisms complement each other and drives the ASS(−) cell toward apoptosis. Importantly, it does not result in an increase in toxicity in normal cells, due to the fact that normal cells possess ASS expression. This combination of ADI-PEG20 with cisplatin is currently in clinical trial. The mechanism(s) of potentiation identified here will be studied in tumor samples from this trial.

## METHODS

### Cell lines

Four ASS (−) melanoma cell lines (A375, Sk-Mel2, A2058 and Mel1220) were used for this study. A375, Sk-Mel2, A2058 were obtained from ATCC without further authentication. All cell lines showed undetectable mycoplasma contamination using routine MycoAlert detection kit (Lonza). Mel1220 was established from a subcutaneous nodule of a patient and has been characterized (9). A375, A2058 and Mel1220 possess BRAFV600E mutation and Sk-Mel2 has N-RAS mutation. A2058 has inducible ASS when treated with ADI-PEG 20 or arginine free media [5]. All cell lines were maintained on EMEM media supplemented with 10% FBS. Mel-114 and Mel-F are primary cultures generated from patients [6]. Mel-F is positive for BRAF mutation while Mel-114 is BRAF wild type. Both primary cultures do not express ASS. A-2058R is a resistant variant of A2058 which express ASS [55]. Mel-GP is a melanoma cell line derived from a patient whose tumor express ASS and did not respond to ADI-PEG20 [6]. These two cell lines were used as negative control. They were also maintained on EMEM. Mel-1220 and Mel-GP express MITF, Melan-A, and tyrosinase.

### Reagents

ADI-PEG 20 was supplied by Polaris Pharmaceuticals Inc. Cisplatin was purchased from clinical pharmacy service. NOXA, MCL1, BCL2, XIAP and SURVIVIN were purchased from Cell Signaling. FANCD2 and FANCL were purchased from Sigma. ADI-PEG20 treated media was prepared by adding ADI-PEG20 at 0.05μg/ml (specific activity 63.5 IU/ml) to EMEM media and incubated for 48 hr. At this concentration there is no detectable arginine in the media while citrulline is >300 μg/ml as detected by HPLC [11].

### Growth inhibitory assay

5×10^4^ cells were seeded in 24 wells plated for 8 hr to allow attachment. Cells were treated with ADI-PEG20 at 0.05ug/ml alone, or with cisplatin(0.1μg/ml) alone, or in combination. At 72 hr. viable cells were counted using trypan blue exclusion. The percentage of viable cells in each dosage was calculated with the control set at 100%. This concentration of ADI-PEG20 was chosen from our previous publication which showed that ADI-PEG20 at 0.05μg/ml is capable of degrading the arginine in EMEM media in 24-36 hr. and no detectable arginine level is found at 72 hr. in all cell lines tested. At 0.1 μg/ml of ADI-PEG20, there is no detectable arginine level in media overnight [11]. This treatment mimics what we observed clinically. However, in cell lines which required RPMI media or DMEM, the concentration of ADI-PEG20 will be higher since they have higher arginine content in the media.

### Apoptotic assay

Annexin-V-Fluos Staining Kit from Roche Applied Science or the Annexin V:FITC Assay Kit (AbD SeroTec) was used to label cells which have been treated with drugs. Briefly, the adherent cells (1.8×10^5^ cells/well seeded overnight) were treated in a 12-well plate. The medium was collected; cells were washed once with DPBS. The wash was combined with the collected medium. The cells were dissociated with 0.25% Typsin/EDTA (Invitrogen) and combined with the collected medium and DPBS. After centrifugation and removal of the supernatant, the cells were washed once with DPBS. Then the cells were processed according to the instruction of the staining kit. The data were collected in an Accuri C6 flow cytometer and analyzed with its bundled CFlow Plus software.

### Immunoblot analysis

Cells were collected in DPBS containing protease (P8340) and phosphatase (P5726) inhibitors (Sigma-Aldrich) at 1:1000 dilutions. The cell pellet was resuspended with 1X lysis buffer (Cell Signaling Technology) plus 1:1000 dilutions of protease and phosphatase inhibitors (same as above) and sonicated briefly on ice. The total protein concentration of the lysate was determined with the Micro BCA Protein Assay Kit from Pierce. The prepared lysate then was mixed with SDS-PAGE sample buffer and heated at 100°C for 10 min. The processed lysate was loaded to SDS-PAGE gel, transferred onto nylon membrane and immunoblotted with various antibodies overnight. This membrane was then blocked with 5% milk (Bio-Rad) in TBS buffer. After incubation with primary antibody and horseradish peroxidase-conjugated secondary antibody, the target protein bands were visualized with SuperSignal West Femto (Thermo-Fisher). The image of bands was captured with a Bio-Rad ChemDoc system. ImageJ was used to estimate the molecular weight, and Quantity One (Bio-Rad) was used to measure the optical density of protein bands detected by western blot.

### Immunofluorescence detection for γH2AX

Cells were seeded onto coverslip in 24-well plate and were treated with cisplatin 0.5 μg/ml in normal media for 48 hr. or in ADI-PEG20 (0.1ug/ml) treated media for 48 hr. The control group consisted of cells grown in normal media and ADI-PEG20 treated media. The coverslip was removed, fixed in methanol, then incubated in 1%BSA / 10% normal goat serum / 0.3M glycine in 0.1% PBS-Tween for 1h to permeabilize the cells and block non-specific protein-protein interactions. The cells were then incubated with the *γH2AX* antibody (ab22551), 10μg/ml and detection was performed using Alexa fluoro 488

Immunoprecipitation Total cell lysate was prepared as above. The cell lysate of all samples were pre-cleaned with isotype antibody (IgG) and gammaBind bead. This process removes nonspecific protein binding to the antibody or to the bead. Following pre-clean process, the specific antibody (anti-Mcl1) and beads were used to bring down Mcl-1 and its interacting protein and then immunoblot with BAK and NOXA.

### Knocking down of SURVIVIN

ON-TARGETplus SMARTpool siRNA targeting human SURVIVIN was purchased from Thermo Scientific. These RNAi molecules are designed to target 4 different regions of SURVIVIN (target sequences: 5′-CAAAGGAAACCAACAAUAA-3′, 5′-GCAAAGGAAACCAACAAUA-3′, 5′-CACCGCAUCUCUACAUUCA-3′, 5′-CCACUGAGAACGAGCCAGA-3′). Transfection of siRNA into cells was done following the manufacturer recommended protocol. At 24 hr post transfection, the transfected cells were seeded onto 24-well plate. ADI-PEG20 or cisplatin alone or combination was added, at the end of 72 hr. Viable cells were counted using trypan blue exclusion.

### Xenograft studies

5×10^6^ of A375 cells was inoculated subcutaneously at the flank of 6 weeks old female Balb/C nude mice. Eight days post inoculation when the tumor size reached about 100 mm^3,^, treatment was started. Mice were separated into four groups (6 mice per each group). The first group (control group) was given saline (10μl/kg). Second group received ADI-PEG 20 53.3 IU/kg IM every 6 days per week for 4 doses. The third group received cisplatin 6 mg/kg I.P once every 6 days for 3 doses and the fourth group received combination treatment. Mice were monitored for body weight, behavior such as mobility, water consumption, eye / hair matting. Tumor size was measured twice weekly using a caliper. The volume was expressed using the formula V=0.5 a x b^2^ where a and b are the short and long diameter respectively. T/C is calculated with T as the median time (in days) required for the treatment group tumor to reach a pre-determined size and C is the median for the control to reach the same size. The T/C value (in percent) is an indication of antitumor effectiveness.

### Gene expression analyses

Briefly, cells seeded in 6-well plates were collected in 0.5 mL of Trizol reagent (Invitrogen) after ADI-PEG20 treatment for 72hrs and total RNA was isolated. Total RNA was quantified with a Nanodrop 8000 Spectrophotometer (Thermo Scientific) and its quality was examined with a Bioanalyzer 2100 using the RNA 6000 Nano kit (Agilent). Biotinylated cRNA was prepared using the Illumina TotalPrep RNA Amplification Kit (Ambion) according to the manufacturer's instructions starting with 400 ng total RNA. Successful cRNA generation was checked using the Bioanalyzer 2100. Samples were added to the Beadchip after randomization using the randomized block design to reduce batch effects. Hybridization to the Sentrix Human-6 Version 2 Expression BeadChip (Illumina), washing and scanning were performed according to the Illumina bead Station 500 manual (Revision C). The resulting microarray data was analyzed using Illumina Bead Studio software.

### Determination of autophagy (Cyto ID staining)

Cells were seeded onto chamber slides and treated with ADI-PEG20 at 0.1 ug/ml, or cisplatin at 0.5 ug/ml or both overnight, wash. The dye based microscopy to detect autophagy was done using the Cyto ID Autophagy Detection kit purchased from Enzo Life Science per manufacture protocol. This green autophagic dye has been shown to be specific for autophagy and co-localized with LC3.

### Statistical analysis

Data in bar graphs were represented as mean ± SD. Student's t-test was used to compare the two sets of data. ANOVA was used to compare three or more set of data. The standard deviation represents three separate experiments. For growth inhibitory and apoptotic assay, each experiment was done in duplicate. Statistical significance was defined as p<0.05 (*) and statistical high significance was defined as p≤0.01.

## SUPPLEMENTARY MATERIAL AND FIGURES


