# Supplementary material for: Targeting argininosuccinate synthetase negative melanomas using combination of arginine degrading enzyme and cisplatin
Source: Oncotarget. 2015 Jan 31;6(8):6295–309. doi: 10.18632/oncotarget.3370 (PMC4467438; doi:10.18632/oncotarget.3370)
Supplement: Supplementary file 1 [file oncotarget-06-6295-s001.pdf]

Targeting argininosuccinate synthetase negative melanomas using combination of arginine degrading enzyme and cisplatin

Supplementary Material

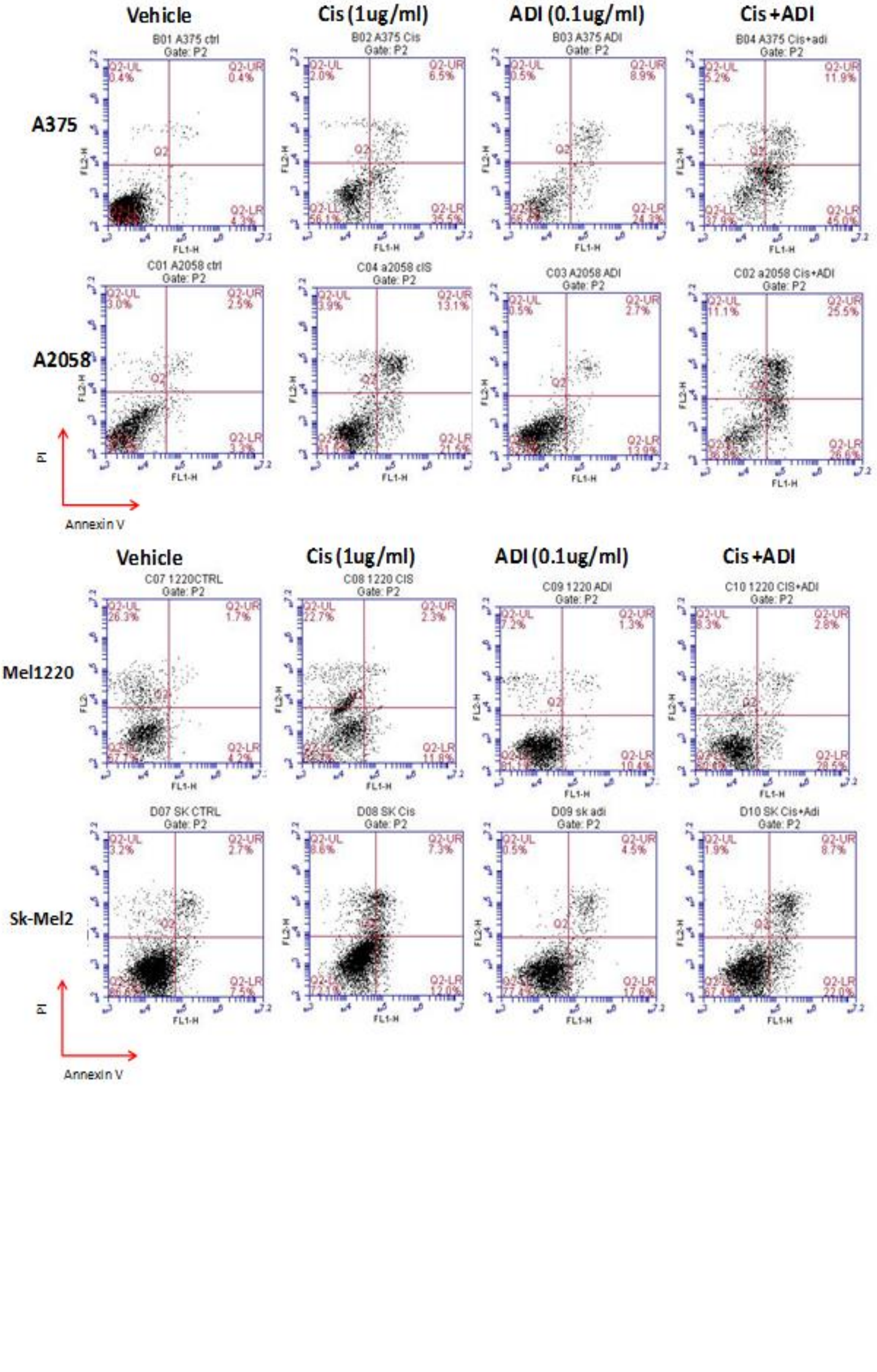

Apoptotic cell death detected by Annexin-V/PI in 4 melanoma cell lines after treated with cisplatin alone, ADI-PEG20, and in combination.

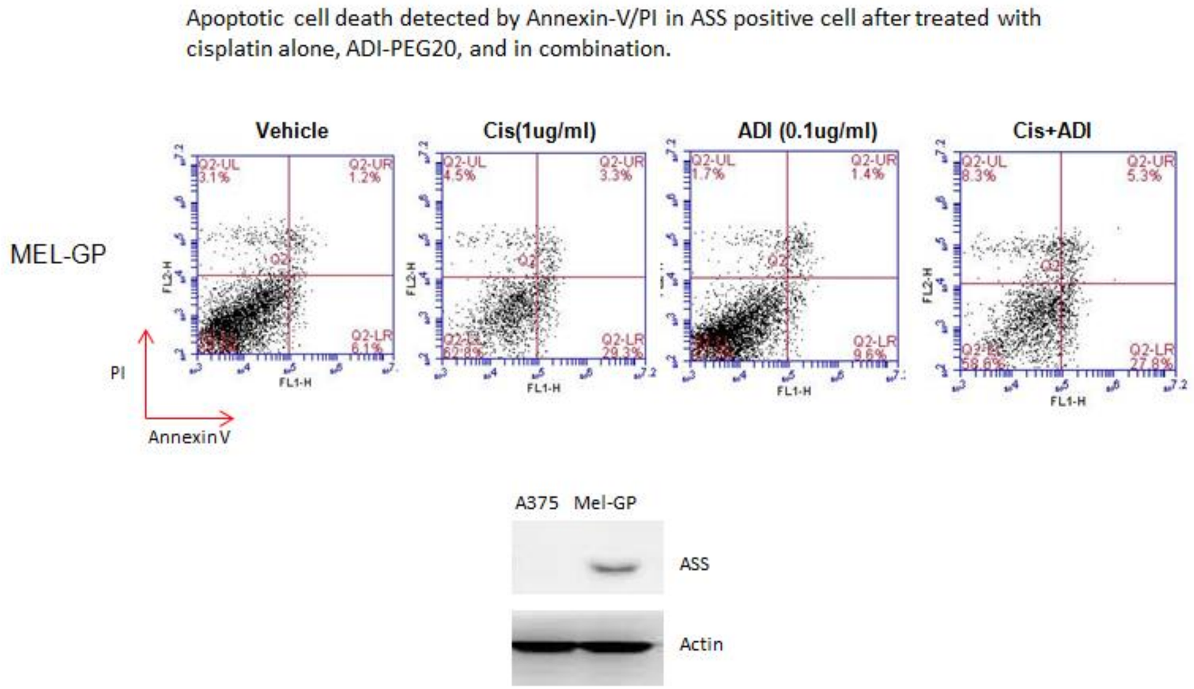

Fig. S1: (a) The percentage of apoptotic cells and total dead cells as detected with Accuri C6 flow cytometry. The horizontal axis represents cells which were positive for Annexin-V (early apoptosis) and the vertical axis represents cells which were positive for PI (cell death). Combination treatment resulted in an increase in the number of cells which were positive for both Annexin-V and P.I. (b) No cell death was observed when treating ASS positive cells (Mel-GP) with ADI-PEG20 alone. There were no significant differences in cell death between cisplatin alone and in combination with ADI-PEG20.

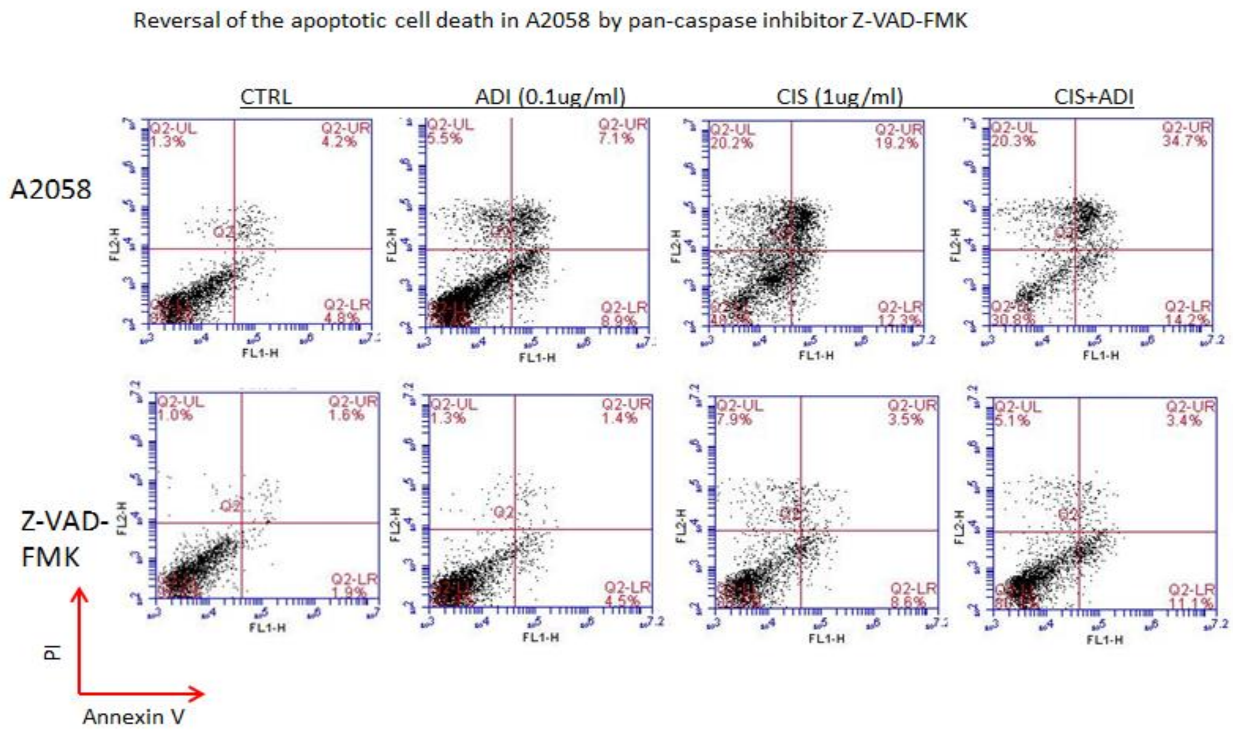

Fig.S2: The percentage of Annexin-V and PI positive after co-treatment with 20µM V-ZAD-FMK (a pan caspase inhibitor). V-ZAD-FMK significantly decreased the number of Annexin-V and PI positive cells, suggesting that these cells undergo caspase dependent apoptotic cell death.

Immunoblot of DNA damage response, anti-apoptotic, and pro-apoptotic proteins in ASS positive cell after treatment with ADI-PEG20 alone, cisplatin alone, and in combination

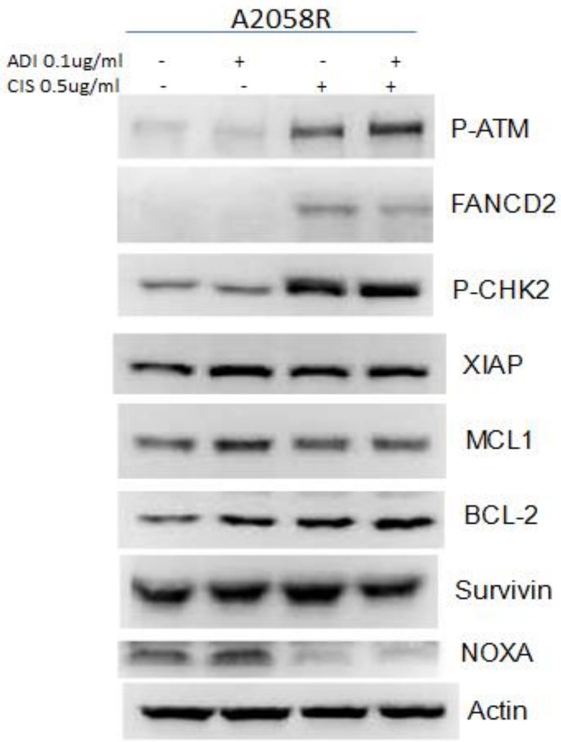

Fig. S3: Immunoblot of DNA repair protein, anti/pro apoptotic protein in ASS positive cell (A2058R). Note: There were no changes after ADI-PEG20 treatment and no changes in the combination treatment when compared to cisplatin alone.

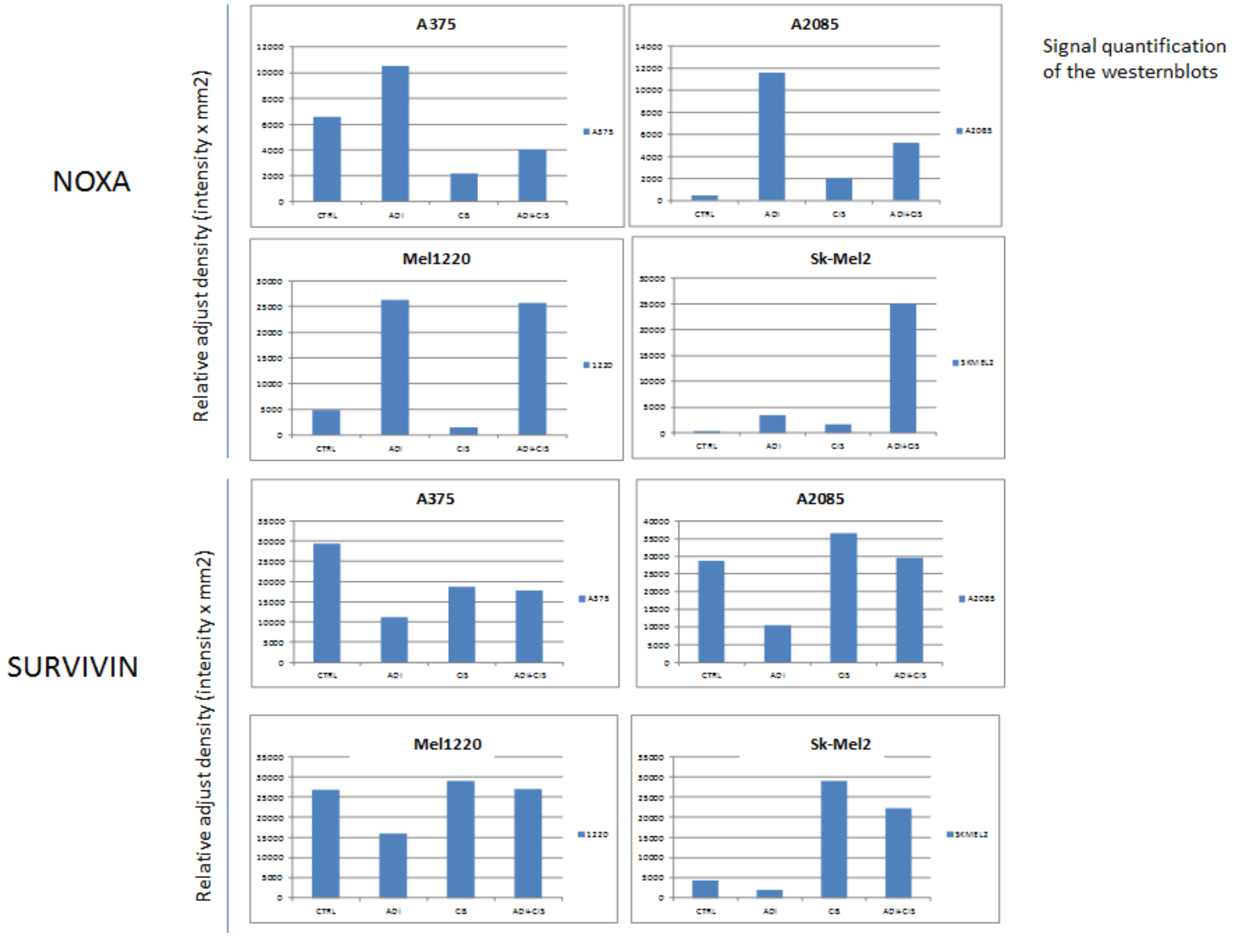

Fig. S4: The signal intensity of immunoblot was quantified using Biorad Quality One with adjustment of actin (as loading control). The bar Graph indicates the relative adjusted density of NOXA and SURVIVIN.

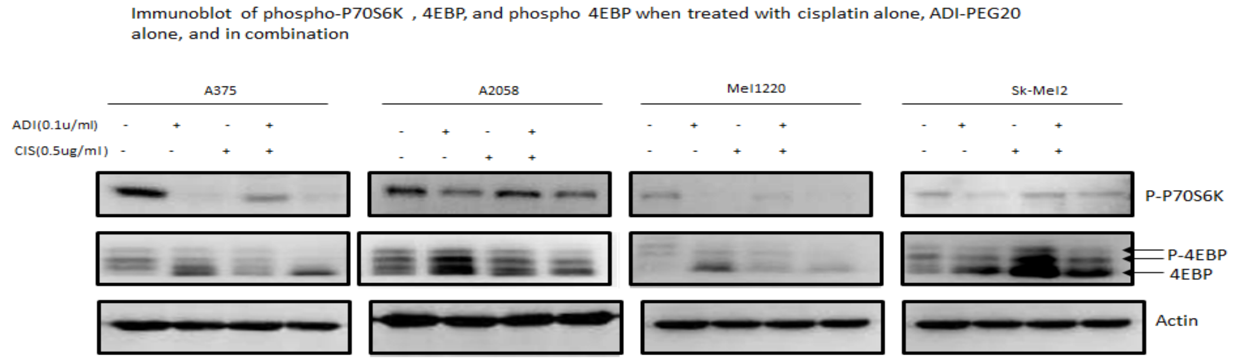

Fig S5: Immunoblot of proteins in the mTOR pathway after ADI-PEG20 alone, cisplatin alone, and in combination. Treatment with ADI-PEG20 resulted in decrease of phosphor-P70S6K and phosphor-4EBP. Cisplatin alone resulted in activation of mTOR surviving pathway as detected by increased phosphor-P70S6K and phosphor-4EBP. However, both phosphor proteins decreased upon combination treatment.

Relative nitric oxide (NO) fluorescent intensity per cell after exposed to ADI-PEG20.

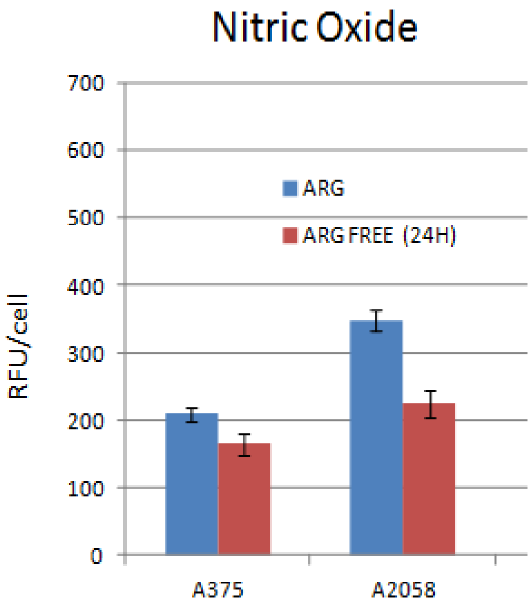

Fig. S6: Relative nitric oxide (NO) fluorescent intensity per cell. Using DAF-2DA probe, we showed that arginine deprivation leads to a decrease in NO production. Bar graph represents the relative fluorescent units/cell via fluorometer plate reader
